# A moderated mediation mechanism underlying the impact of website telepresence on purchase intention — Evidence from Chinese female college student customers

**DOI:** 10.3389/fpsyg.2022.902414

**Published:** 2022-09-02

**Authors:** Guiqin Zhu, Shuaihe Jiang, Kai Li

**Affiliations:** ^1^School of Education, Chongqing Normal University, Chongqing, China; ^2^School of Law and Sociology, Xinyang Normal University, Xinyang, China; ^3^School of Management, Xi’an University of Architecture and Technology, Xi’an, China

**Keywords:** website telepresence, flow experience, socioeconomic status, purchase behavior, consuming psychology

## Abstract

Telepresence in e-commerce, the feeling of resembling shopping in a physical store, plays a critical role in determining online purchase intention. However, the cognitive mechanism and boundary conditions about its effect still need further investigation. The current study construed flow experience and socioeconomic status as important variables and developed a moderated mediation model for their roles in the effect of telepresence. The model was supported by our study where a group of Chinese female college students participated in simulated online apparel shopping and completes relevant questionnaire surveys. The results show that: (1) website telepresence predicts positively the purchase intention of females, (2) flow experience mediates the impact of website telepresence on purchase intention, and (3) the relationship between website telepresence and flow experience could be moderated by socioeconomic status, namely, females with higher socioeconomic status demonstrate stronger mediation of flow experience. These findings can help researchers and online retailers understand the flow concept in e-commerce and formulate marketing strategies to retain consumers with different socioeconomic statuses.

## Introduction

E-commerce platforms scramble to enhance website telepresence in hope to attract and maintain consumers’ attention (Hausman and Siekpe, 2009). It has been well-established that telepresence, the psychological state of “being there” and the feeling of resembling shopping in a physical store, is pivotal for users’ immersion into online shopping. It affects customer attitudes toward the service/product and ultimately purchases intention (Hassanein and Head, 2007; Lee, 2018). Despite the significance, explanatory frameworks about the mechanism of its impact are still vague (Darley et al., 2010; Tang et al., 2021), due to the complexity of customer behaviors and the ever-evolving internet technologies. Researchers have, therefore, called for more studies on potential mediators and moderators, as well as their interaction to explain the telepresence effect on online shopping (Darley et al., 2010; Gao et al., 2018; Tang et al., 2021).

Over the past decades, flow experience has been regarded as an important factor influencing shopping behavior (Koufaris, 2002; Obadã, 2013; Liu et al., 2020). Flow experience refers to an optimal state where people devote all their attention to the task at hand and experience a deep sense of enjoyment and fulfillment (Csikszentmihalyi, 1988; Csikszentmihalyi and Csikzentmihaly, 1990; Abuhamdeh, 2020). Originally developed to account for activities such as reading, music, invention, it has been extended to and highly valued in e-commerce. Flow experience affects consumer online information searching behaviors (Mathwick and Rigdon, 2004; Wang and Wang, 2020) and has been associated with positive perceptions and attitudes toward website browsing (Shim et al., 2015), brand loyalty (Zhou et al., 2010; Shim et al., 2015; Su et al., 2016) as well as purchase intention (Ozkara et al., 2017). These findings have well-established flow experience as a major psychological construct accounting for the impact of telepresence on shopping behaviors.

Despite this, till now little has been known about the boundary conditions for the mediation of flow experience (Wang and Wang, 2020). In the current study, we decided to investigate its susceptibility to socioeconomic status (SES), a prominent demographic variable for company marketing. When formulating marketing strategies and product promotion, socioeconomic status factors such as income and occupation constitute the primary concerns because they determine buyers’ purchasing power and technology accessibility. SES has been found to determine critically consumers’ shopping behavior in a multitude of studies (Pechey and Monsivais, 2015; Imtiaz et al., 2019; see a review: Akar and Nasir, 2015). However, there are some other studies reporting that the socioeconomic characteristics of the individuals do not have a significant impact on the behavior of e-shoppers (Hernández et al., 2011; Bringula et al., 2018; Vajjhala and Strang, 2019), leading to suggestions that e-commerce industries must turn their attention away from socioeconomic variables (Hernández et al., 2011). Meanwhile, though SES and flow experience are among the most valued variables in consumer behavior research, it is surprisingly interesting that their interaction has been sparsely discussed in e-commerce archives. Our literature searching (up to June 28, 2022) in global databases such as https://www.sciencedirect.com/, https://pubmed.ncbi.nlm.nih.gov/, Google Scholar using the combined keywords of (*“socioeconomic status” AND “Flow experience”)* or (*“socioeconomic status” AND “Flow”)*, as well as the CNKI^1^ in mainland China using corresponding Chinese keywords, yielded very limited empirical articles on their relationship between SES and flow experience in online shopping.

In light of this, the present study aimed to explore the role of flow experience underlying the impact of website telepresence on purchase intention, especially how the socioeconomic status of e-shoppers would moderate the mediation effect. We included purchase attention as the dependent variable because of its significantly positive influence on online shopping behavior (Lim et al., 2016; Wang and Wang, 2020). Answers to these questions can help us delineate how flow experience functions in e-commerce circumstances and contribute to the flow theory. They are also expected to provide insights into adjusting marketing and advertisement strategies for e-commerce practitioners.

## Literature review and research hypotheses

### The influence of website telepresence on purchase intention

When shopping in a brick-and-mortar store, consumers can feel directly the commodities (such as touching clothes, tasting food, etc.) and are more likely to indulge in the purchase process. E-commerce, in contrast, is supported by network technology and largely intangible (Darley et al., 2010; Wang and Wang, 2020). In online shopping, consumers get access to information about the commodities of interest mainly by searching on the internet. On websites with high vividity and co-creation, the customers can easily get more information about prices, sales records, and feedback comments on the product of interest, they can easily make comparisons among different products or even products across different shopping agents (Dang et al., 2020). On websites with advanced services, the customers can chat freely with the sales agents and with companion buyers. These features enable the customers to accumulate adequate knowledge about the products and develop trust and social bonds as they were shopping in brick-and-mortar stores, which are important for attracting and maintaining the attention of the consumers (Dang et al., 2020).

Currently, increasing e-commerce platforms are enhancing website telepresence to attract consumers’ attention. Website telepresence refers to the feeling of resembling shopping in a physical store during online shopping, in other words, consumers can have a similar feeling of shopping in physical stores when visiting the shopping website (Coyle and Thorson, 2001). Website telepresence involves mainly two important aspects (Coyle and Thorson, 2001). The first one regards how commodity information is presented on shopping websites, for example, by using pictures, videos, etc. The second one regards how the consumers can interact with the websites. Therefore, there are two critical dimensions of website telepresence: vividness and interactivity (Coyle and Thorson, 2001), which are frequently manipulated in previous experimental studies (Fiore et al., 2005; Fortin and Dholakia, 2005). The website telepresence has also been found beneficial to increase consumers’ trust in virtual stores (Hassanein and Head, 2007), which constitutes a prerequisite for purchasing behavior (Darley et al., 2010). Particularly, the interactivity between the website and consumers, the style of the website interface, and relevant factors are stimulating purchase behavior (Darley et al., 2010). Higher website telepresence can enhance consumers’ happiness and increase loyalty to the online store (Jarvenpaa and Todd, 1996). When consumers have a feeling of a high level of telepresence in a virtual shopping environment, they would have stronger purchase intention (Gao et al., 2018). Based on this, we propose the first hypothesis:

*Hypothesis 1 (H1): website telepresence would have a positive effect on purchase intention.*

### Mediation of flow experience

An essential function of website telepresence is to improve consumers’ shopping experience. People shop not merely for possessing commodities, but frequently as a way of leisure and entertainment. Browsing websites and purchasing goods online give some customers pleasure and distracted them from chores. Previous studies have shown that shopping in an experiential way has more positive emotional experiences than traditional purchases in physical stores. For instance, although possessing items can bring happiness, happiness generally lasts only for a short time (Richins, 2012). In contrast, the positive feeling brought by purchasing online could be more lasting than pure shopping in a physical store (Carter and Gilovich, 2014).

Flow experience refers to such a kind of positive emotion accompanied by a pleasant experience, which is characterized by focus and devotion to the goal at hand (Csikszentmihalyi, 1988). This emotional experience is sustainable, just like a constant “water flow” (Csikszentmihalyi, 1988). Flow experience makes individuals forget the existence of time and space (Csikszentmihalyi, 1988). It occurs frequently in creative activities (such as art, music, writing, etc.) and leisure activities (such as dancing, rock climbing, surfing, etc.) (Harmat et al., 2016). With the advancement of internet technology and the rapid development of online shopping, the impact of shopping experience has been regarded as an important psychological factor influencing online purchase behavior. Therefore, it is necessary to study this pleasant experience generated by consumers during website browsing (Harmat et al., 2016).

Previous studies have found that flow experience could positively predict online consumers’ brand loyalty (Wu et al., 2010; Zhou et al., 2010; Su et al., 2016) and purchase intention (Korzaan and Boswell, 2008; Hausman and Siekpe, 2009). For instance, the perceived pleasure during the online shopping process has a positive effect on consumers, which will be helpful for them to form a positive impression and lead them more willing to seek relevant information about product and service (Koufaris, 2002). It was further found that if consumers experience higher level of telepresence, they are more likely to indulge in virtual shopping and show stronger purchase intention (Fortin and Dholakia, 2005; Wang et al., 2017). Meanwhile, the flow experience during online shopping could be enhanced by a beautiful and interactive website platform, fast response from the sellers or service agents and rapid delivery, etc. (Park and Kim, 2003). Website telepresence can satisfy consumers’ psychological needs such as sensory experience, leisure, and entertainment, and can enhance consumers’ shopping experience (Dholakia and Zhao, 2009), thereby effectively improving the flow experience (Wang and Lei, 2017). Website telepresence also enhances the fantasy when browsing the website, which in turn enhances the consumer’s shopping pleasure (Song et al., 2007). To sum up, these findings show that a high level of telepresence enhances a pleasant shopping experience and flow experience, in turn, increases shopping intention, suggesting that flow experience may act as a mediator for the impact of those two factors. We, therefore, come up with the second hypothesis:

*Hypothesis 2 (H2): flow experience may mediate the relationship between website telepresence and females’ online shopping intention.*

### The moderated mediation of socioeconomic status

There are many factors affecting online shopping, such as the characteristics of e-commerce sellers, consumers, product, marketing, etc. (Wang and Lei, 2013). The generation of flow experience is not only dependent on website factors but may also be subject to consumer characteristics. Different from shopping in a physical store, the commodities in online cannot be touched, which increases the risks of product defects and low quality of post-sales service (Javadi et al., 2012). Higher level of risk perception would in turn affects online shopping intention (Akhter, 2012). Moreover, previous studies have established that social status exerts a substantial impact on risk perception: compared with consumers in the upper class, the individuals in the lower class probably demonstrate stronger risk perception. For instance, consumers of the lower socioeconomic status are characterized by more external threats and less control resources compared with the consumers of the higher status (Pickett and Gardner, 2005). People in lower-class also show more threat-related physiological reactions (Evans et al., 2010), and they feel more threatened, hostile, and show a higher heart rate when exposed to blurred videos (Chen and Matthews, 2001). People in lower class also self-report less trust in others (Gallo and Matthews, 2003). Simultaneously, reducing risk perception makes the consumers indulge more in online shopping (Wang et al., 2017), and consumers with higher income and consumers with higher education levels can get more happiness from the experience of purchase (Van Boven and Gilovich, 2003). People with low socioeconomic status value physical possession more than the experience of shopping. It is more difficult for them to experience the happiness of online shopping, which in turn may inhibit purchase intentions (Thomas and Millar, 2013).

It is worth noting that there are also some alternative findings that socioeconomic characteristics do not have a significant impact on online shopping behavior (Hernández et al., 2011; Bringula et al., 2018; Vajjhala and Strang, 2019). For instance, Hernández et al. (2011) found socioeconomic variables such as age, gender, and income did not condition purchasing behaviors of experienced e-shoppers. Similar results were also reported that income level had nothing to do with the willingness to buy smartphones online among American and Indian senior bachelor level students (Vajjhala and Strang, 2019) and Philippine college students (Bringula et al., 2018). These studies have led to the suggestions that internet was equal for all consumers and socioeconomic variables could be scrapped from the consideration of e-venders (Hernández et al., 2011). Meanwhile, though SES and flow experience are among the most valued variables in consumer behavior research, it is surprisingly interesting that their interaction has been sparsely discussed in e-commerce archives. So, there is a necessity to explore their relationship empirically. However, given the majority of previous reports of a positive association between SES and shopping behaviors, we proposed that compared with people with low economic status, those with high economic status might have more flow experience from website telepresence. So, our third hypothesis is:

*Hypothesis 3 (H3): Socioeconomic status moderates the effect of website telepresence on purchasing intention and flow experience.*

**FIGURE 1:**
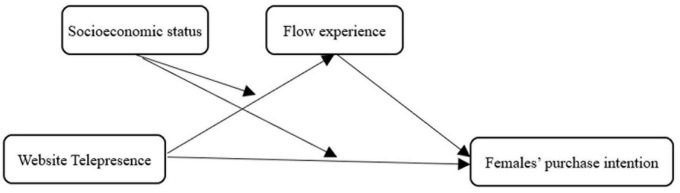
Conceptual model of this study.

Since flow experience is hypothesized to mediate the impact of telepresence on shopping intention (H2) and the relationship between telepresence and flow experience is hypothesized to be moderated by socioeconomic status (H3), if both H2 and H3 get proved, we could probably envision that socioeconomic status can reshape the mediating effect of flow experience. We, therefore, hypothesize a moderated mediation mechanism that (Figure 1):

*Hypothesis 4 (H4): The mediating effect of flow experience on website telepresence and purchase intention may be moderated by socioeconomic status. Specifically, the mediating effect of flow experience in groups with high socioeconomic status is stronger than that in those with low socioeconomic status.*

### Purpose of the current study

We tested these hypotheses by a simulated online purchase task and questionnaire surveys on a group of Chinese female college students. The participants were asked to purchase apparels *via* a simulated website advertising the clothes which varied in their interactivity and vividity. They were then asked to complete questionnaires about relevant measurements, the relationship among which was explored using the Macro Process in SPSS. Only female participants were included because female consumers are increasingly important in China’s prospering e-commerce sector and they enjoy larger decision rights in household purchasing like apparels. For instance, a report in March 2021 by the China e-commerce giant JD suggests that as many as 75 percent of household purchasing decisions were made by women (He, 2021). The results could help us investigate the roles of flow experience and socioeconomic status behind the telepresence effect, at least in the current context. Limitations of the absence of male consumers will be discussed later in the current study.

## Methods

### Participants

The present study randomly selected 118 female college students for participation, with an average age of 20.94 ± 2.41.

### Instruments

#### Website telepresence scale

There are a total of five items for website telepresence (Fiore et al., 2005), such as “the website provides me with information (size, color, details, etc.) that I can get in physical stores.” Seven-point semantic scoring method is adopted (1 = completely disagree, 7 = completely agree). The higher the score is, the higher the level of website telepresence. In this study, the Cronbach’s coefficient of this scale was 0.76.

#### Flow experience scale

The flow experience scale proposed by Webster et al. (1993) was adopted, (Webster et al., 1993), namely, four dimensions (i.e., control, focus, curiosity, and pleasure) with three items in each dimension, such as “Interaction with this website makes me curious” and so on. A seven-point semantic scoring method was used (1 = completely disagree, 7 = completely agree), with higher scores indicating higher intoxication. In this study, the Cronbach’s coefficient of the scale was 0.86.

#### Socioeconomic status scale

Socioeconomic status is divided into objective and subjective socioeconomic status. Objective socioeconomic status is mainly measured by education level, income level, and educational background, while subjective socioeconomic status refers to an individual’s perception of her/his socioeconomic standing (Huang et al., 2017). Goodman et al. (2003) held that subjective socioeconomic status could capture more accurately the sensitivity of subjective perception in social status and provide more valuable reference than objective indicators (Goodman et al., 2003). In the current study, the participants were presented with a ladder with 10 levels, which represented the perceived social status of an individual. The higher the level of the ladder, the higher the socioeconomic status of the individual (Adler et al., 2000).

#### Online purchase intention scale

We adopted the measurement items compiled by Wang et al. (2017). They compiled these measurement items according to the characteristics of consumers’ actual online shopping environment (Wang et al., 2017). There are three items in total, such as “If I need to buy clothes online, I will consider buying on this website.” Using a Likert scale (seven-point evaluation) (1 = completely disagree, 7 = completely agree), higher scores indicate stronger purchase intentions. In this study, the Cronbach’s coefficient of the scale was 0.93.

### Procedure

The participants were randomly assigned to a level of website telepresence. Applying research materials used by Wang et al. (2017), we manipulated the levels of website telepresence according to vividness and interactivity (see Supplementary Appendix 2 for example websites). Specifically, we took females’ clothing as the commodities of interest and designed two virtual clothing shopping websites, one with high website telepresence and the other with low website telepresence. A high level of telepresence meant that the website’s vividness and interactivity were both at high levels. On the contrary, a low level of telepresence suggests that the website’s vividness and interactivity are both at low levels. Fifty-eight participants were assigned randomly to the high telepresence website and the other 60 participants to the low telepresence website.

Finally, we then asked all the participants to fill in the survey which included the questionnaires on website telepresence, flow experience, purchase intention, and subjective socioeconomic status (see Supplementary Appendix 1 for the items of these questionnaires). The survey also included some other information such as answering earnestness, monthly living expenses, etc. Independent-sample *T*-test shows that the ratings of website telepresence demonstrate a significant difference between the two participant groups [*M ± SD*_high_ = 5.076 ± 0.949, *M ± SD*_low_ = 3.787 ± 0.944, *t*(116) = 7.395, *p* < 0.001], which suggests the validity of web design as the manipulation of the independent variables of website telepresence. The earnestness [*M ± SD*_high_ = 5.483 ± 1.064, *M ± SD*_low_ = 5.20 ± 0.953, *t*(116) = 1.522, *p* = 0.131] and monthly consumption level [*M ± SD*_high_ = 2.775 ± 1.027, *M ± SD*_low_ = 2.783 ± 1.166, *t*(116) = –0.037, *p* = 0.971] demonstrate no significant group differences.

## Results

### Common method bias

The data were tested for common method bias using one-factor model constructed by Harman. In this test, the un-rotated principal component analysis (PCA) was performed involving all variables. The results revealed six factors with eigenvalues greater than 1, and the first factor extracted explained 36.30% of the total critical value, which was lower than the critical value of 40%, suggesting no significant common method bias.

### Mean value, standard deviation, and correlation matrix of each variable

We could see from Table 1 the mean, standard deviation, and Pearson’s correlation among the variables. The analysis showed that flow experience correlated positively with website telepresence (*r* = 0.669, *p* < 0.001) and purchase intention (*r* = 0.685, *p* < 0.001). There was also significantly positive correlation between website telepresence and purchase intention (*r* = 0.698, *p* < 0.01). There was no significant correlation between socioeconomic status (SES) and flow experience (*r* = –0.026, *p* = 0.781), nor between SES and purchase intention (*r* = 0.018, *p* = 0.844), nor between SES and purchase intention (*r* = 0.004, *p* = 0.967). There was significant impact of students’ major on participants’ purchase intention [*F*_(2,115)_ = 4.257, *p* = 0.016], but not on website telepresence [*F*_(2,115)_ = 1.527, *p* = 0.222], flow experience [*F*_(2,115)_ = 0.851, *p* = 0.430], and rating of SES [*F*_(2,115)_ = 0.136, *p* = 0.873]. There was no significant impact of students’ grade on telepresence [*F*_(4,113)_ = 2.112, *p* = 0.084], purchase intention [*F*_(4,113)_ = 1.758, *p* = 0.142], flow experience [*F*_(4,113)_ = 1.716, *p* = 0.151], and rating of SES [*F*_(4,113)_ = 0.368, *p* = 0.831].

**TABLE 1:** Mean, standard deviation, and correlation matrix of each variable (*n* = 118).

	1	2	3	4	5	6	7	8
1. Website telepresence	—							
2. Flow experience	0.669***	—						
3. SES	–0.004	0.026	—					
4. Purchase intention	0.698***	0.685***	0.018	—				
5. Monthly expenditure	−0.156	–0.034	0.299**	-0.034	—			
6. Task earnestness	0.364***	0.510***	0.118	0.300**	-0.063	—		
7. Age	0.059	0.078	–0.045	0.057	0.131	0.09	—	
8. Years e-shopping	–0.022	–0.087	0.298**	–0.044	0318**	–0.026	0.118	—
M	4.420	4.282	5.017	4.071	2.780	5.339	20.941	8.877
SD	1.144	0.916	1.240	1.576	1.095	1.015	2.412	2.656

*p < 0.05, **p < 0.01, ***p < 0.001, ns: not significant (the same applies hereinafter).

### Test for the mediating effect of flow experience

**FIGURE 2:**
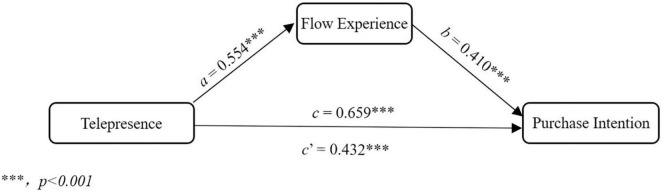
The mediation of flow experience. Flow experience mediates significantly the relationship between website telepresence and purchase intention.

We then explored the mediating effect of flow experience behind the relationship between website telepresence and purchase intention by the Process macro for SPSS (Hayes, 2018). Confounding variables, namely, age, major, grade, monthly expenditure, task earnestness, and years of online shopping experience were all included as covariates of uninterest in the mediation model. Major and grade were converted into dummy variables, and all other predictors were standardized to *Z*-scores before entering into the process as suggested by Wen and Ye (2014). The significance of the mediation effect was determined by deviation-corrected non-parametric percentile bootstrapping (*n* = 5000). We can see from Figure 2 that when flow experience is not taken into account, website telepresence predicts significantly purchase intention [the regression coefficient for overall effect, *c* = 0.659, *t* = 8.794, *p* < 0.001, interval of 95% confidence: (*CI*: 0.523, 0.794)]. When flow experience is included as the mediating variable, website telepresence still predicts purchase intention significantly [*c’* = 0.432, *t* = 5.049, *p* < 0.001, (*CI*: 0.262, 0.602)], but to a less degree. The decrease is due to the significant prediction of telepresence on flow experience [*a* = 0.554, *t* = 7.557, *p* < 0.001, (*CI*: 0.528, 0.799)] and the prediction of flow experience on purchase intention [*b* = 0.410, *t* = 4.484 *p* < 0.001, (*CI*: 0.229, 0.591)], the combination of which results into a significant indirect mediating effect of flow experience [effect size = 0.227, (*CI*: 0.116, 0.374)].

### The moderation of socioeconomic status on the mediation of flow experience

**FIGURE 3:**
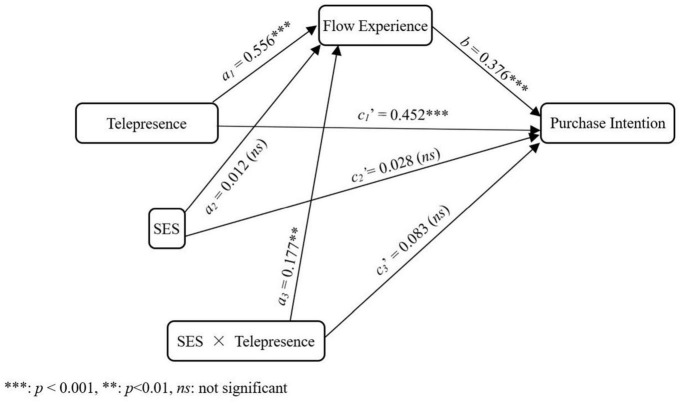
The moderated mediation model underlying the effect of telepresence on purchase intention.

A moderated mediation model (model number: 8) in PROCESS macro was used to test hypothesis 4. Confounding variables mentioned in 4.3 were all included as covariates of uninterest and treated similarly in model estimation. As shown in Figure 3, though flow experience is not predicted by SES [*a*_2_ = 0.012, *t* = 0.193, *p* = 0.862, (*CI:* –0.126, 0.151)], it is positively predicted by websites telepresence [*a_1_* = 0.556, *t* = 7.805, *p* < 0.001, (*CI:* 0.415, 0.698)], and positively predicted by the interaction between website telepresence and SES [*a_3_* = 0.177, *t* = 2.861, *p* = 0.005, (*CI:* 0.054, 0.300)]. Meanwhile, though purchase intention is not predicted by SES [*c*_2_′ => 0.028, *t* = 0.411, *p* = 0.682, (*CI*: –0.106, 0.162)], nor by the interaction between website telepresence and SES [*c*_3_′ = 0.083, *t* = 1.326, *p* = 0.188, (*CI:* –0.041, 0.206)], it is positively predicted by websites telepresence [*c*_1_′ = 0.452, *t* = 5.198, *p* < 0.001, (*CI:* 0.280, 0.625)] and flow experience [*b* = 0.376, *t* = 3.959, *p* < 0.001, (*CI:* 0.188, 0.565)]. Importantly, the analysis reveals significant moderated mediation involving flow experience [*effect size* = 0.067, (*CI:* 0.019, 0.134)].

**FIGURE 4:**
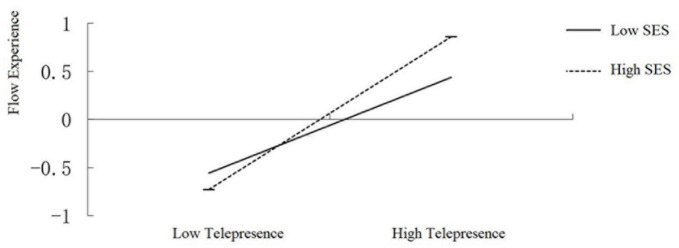
The slope diagrams about moderating effect of socioeconomic status.

We then constructed slope diagrams to visualize the moderating effect of socioeconomic status. To this end, we divided the participants into high and low SES groups based on –1 and 1 standard deviation from the group mean. The results showed that website telepresence predicted significantly positively flow experience both in in the low socioeconomic status group (β = 0.50, *t* = 5.71, *p* < 0.001) and the high socioeconomic status group (β = 0.79, *t* = 9.82, *p* < 0.001) (Figure 4). However, the moderated mediation revealed in the past paragraph suggests that telepresence has a significantly larger impact on flow experience in the high SES group (*effect* = 0.535) than in the low SES group (*effect* = 0.369), which means the effect of website telepresence on them is significantly enhanced with the improvement of females’ socioeconomic status.

We also analyzed the moderation of socioeconomic status on the relationship between website telepresence and purchase intention. This revealed that the mediation of flow experience was significant both in females with high [mediation effect = 0.276, (*CI*: 0.129, 0.460)] and low socioeconomic statuses [mediation effect = 0.143, (*CI*: 0.057, 0.275)]; however, the mediation of flow experience was significantly more enhanced along with the rising in socioeconomic status.

To explore the reliability of these findings, especially factors such as collinearity, homoscedasticity, and normality would potentially affect our results, we re-tested the moderated mediation model by structural equation modeling (SEM) using the MPlus software (Muthén and Muthén, 2017). SEM can help manage the effects of random measurement error. It treats mediation or moderation as a model instead of procedure and estimates the coefficients simultaneously and iteratively using maximum likelihood. There are concerns that factors such as the complexity of the models, the degree of unreliability in measurement, and the correlation between the variables in the model could contribute to random measurement error, which might bias to some extent the results of OLS regression that PROCESS relies on (Hayes et al., 2017). Specifically, we hypothesized that SES, telepresence, and the interaction between SES and telepresence (intST) would predict flow experience, while flow experience, telepresence, and intST would predict purchase intention (Supplementary Appendix 3). We set a number of bootstrapping to 5000 and included all the covariates as in the PROCESS testing. The test revealed strong evidence against the baseline model, χ^2^ *_(baseline)_* = 221.577, *df* = 27, *p* < 0.001. CFI = 1.000, TLI = 1.000, SRMR < 0.001. As shown in Figure 3, though flow experience is not predicted by SES [β = 0.012, *t* = 0.193, *p* = 0.847, (CI: –0.121, 0.138)], it is positively predicted by websites telepresence [β = 0.556, *t* = 7.735, *p* < 0.001, (*CI*: 0.401, 0.691)], and positively predicted by the interaction between website telepresence and SES [β = 0.189, *t* = 3.077, *p* = 0.002, (*CI*: 0.054, 0.299)]. Meanwhile, purchase intention is not predicted by SES [β = 0.028, *t* = 0.417, *p* = 0.677, (*CI:* –0.107, 0.155)], nor by the interaction between website telepresence and SES [β = 0.088, *t* = 1.409, *p* = 0.159, (*CI:* –0.029, 0.218)], but it is positively predicted by websites telepresence [β = 0.452, *t* = 4.860, *p* < 0.001, (*CI:* 0.272, 0.635)] and flow experience [β = 0.376, *t* = 3.872, *p* < 0.001, (*CI:* 0.178, 0.562)]. These observations were consistent with those from the Process macro.

## Discussion

Inspired by the flow experience theory, the current study explored the influence of website telepresence on purchase intention, particularly the roles of flow experience and socioeconomic stats behind this effect by a moderated mediation model. Based on a group of Chinese female college students, our study identified a significant mediating effect of flow experience behind the role of telepresence on purchase intention; we also found that the mediating effect of flow experience was moderated by socioeconomic status. These results extend our understanding of the mechanism of flow experience in online shopping, and demonstrates valuable implications for online vendors to make marketing strategies and promote their products.

Our study replicated several findings which have been reported in the literature. First, the hypothesis was supported as we found that website telepresence positively predicted purchase intention when Chinese college female students were shopping online for clothes. This is consistent with the reports such as (Hassanein and Head, 2007; Gao et al., 2018; Lee, 2018; Ye et al., 2020) which pinpoint the importance of telepresence in e-commerce. Second, we identified the mediation of flow experience behind the impact of telepresence, which is in line with the widely acknowledged role of flow experience in previous studies (Shim et al., 2015; Wang et al., 2021) and supports our hypothesis 2. Customers’ loyalty depends on whether online shopping satisfies their psychological needs. Studies have found that compared with physical purchase, experiential purchase can bring consumers a more pleasant experience and higher satisfaction (Caprariello and Reis, 2013; Thomas and Millar, 2013). Consumers tend to incorporate the unique feeling brought by the flow experience of online shopping into their self-awareness, and thus multiplying the degree of their meaningful experience (Howell and Hill, 2009; Carter and Gilovich, 2012). Previous studies have reported that in virtual reality and website-based online platforms, characteristics like co-creation, intractability can induce the state of flow and improve brand attitudes and purchase intentions (Park and Kim, 2003; Cowan and Ketron, 2018). In live e-commerce, host charm, trust in the host and social presence significantly affect the flow experience, which in turn determines consumers’ purchase intention (Wang et al., 2021).

Some previous studies report that higher income is associated with higher risk-taking propensity which may result in higher levels of comfort while shopping online (Fiore et al., 2005). In contrast, customers with lower income levels feel more external threats (Evans et al., 2010) but less control over resources (Chen and Matthews, 2001; Pickett and Gardner, 2005) or trust (Gallo and Matthews, 2003), discouraging online shoppers from online transactions because of the fear of potential financial losses (Fiore et al., 2005). Since trust (Zhou, 2012) and self-efficacy (Peifer et al., 2020) are positively associated with flow experience, these findings seem to suggest socioeconomic status may be positively associated with the flow experience during online shopping. However, as stated in the introduction and literature review, despite both of them being among the most frequently investigated variables in consumer behavior research, currently there have been limited studies directly exploring their relationship in the e-commerce context. Our study bridged this gap in the literature by exploring the direct relationships between flow experience and demographic profiles of consumers.

Contrary to this prediction, we did not find a significant direct relationship between socioeconomic status and purchase intention, and hypothesis 3 was not supported. We did not observe a correlation between socioeconomic status and flow experience. These observations may be consistent with the results by Hernández et al. (2011) where the socioeconomic variables such as age, gender, and income did not demonstrate a significant impact on purchasing behaviors of experienced e-shoppers. They may also be consistent with the findings by Vajjhala and Strang (2019) reporting no impact of age, gender, and income levels on the e-commerce behaviors of American and Indian senior bachelor level students. There are also reports that though Philippine college students are economically capable of buying smartphones, their monthly family income has no on their online shopping for smartphones (Bringula et al., 2018). However, our observations are not inconsistent with other studies which reported important roles of SES variables on online shopping behaviors. For instance, there are many studies showing that income level has shown a significant positive association with online purchasing attitude and intention (Akman and Rehan, 2014; see a review: Akar and Nasir, 2015). Consumers who have higher income levels are more likely to shop online than those who have lower income levels (Park and Kim, 2003; Hernández et al., 2011; Punj, 2011, 2012; Gong et al., 2013). In a comprehensive literature review, Akar and Nasir (2015) listed several demographic variables which impact online shopping behaviors or purchase intention, and SES variables like age, education, culture, occupation, credit card usage, and income levels are among the most highlighted.

One possible cause for our failure to observe a direct impact of income on flow experience and purchase intention would be participants’ sampling bias. Our study recruited female college students only as respondents. As could be seen in the past paragraph, all the four studies which did not observe a significant impact of SES variables recruited college students as participants. One may speculate that this narrow spectrum of participants would lead to an insufficient variation of critical variables such as SES and ultimately insensitive statistical coefficients. However, we would like to venture that though only female college students were recruited, the ratings of subjective SES ranged from 2 to 8, which covered most of the spectrum of the SES ladder scale (10 rungs). The mean SES was 5.017 and the standardized variation was 1.240. Among the seven-point Likert scales, the range of mean purchase intention was between 1 and 7, the range of telepresence was from 1 to 6.8, and that of mean flow experience was between 2 and 6.25. This means that the independent variable SES has a variance that may enable us to get reasonable statistical, especially correlation and regression coefficients. Nevertheless, the authors should still be cautious about the limitation of our participants’ sampling. Though college students are more diversified and an increasing number of Chinese females attend university, in many universities or majors the females even outnumber their male counterparts, currently not all people can attend university because they have to attend high school first and then pass college entrance examination which tests only on intelligence and knowledge but also financial resources and social support. According to the Chinese news outlet Xinhua, the gross enrollment rate in tertiary education in China in 2021 reached 57.8% of the respective age cohorts (Xinhua, 2022). That means female college students constitute a special group of their peer population. According to the social identity theory, group membership can help people to instill meaning in social situations, and help them to define who they are, how they think of themselves and others and to determine how they relate to others (Tajfel and Turner, 2004) and even shopping behavior (Woodruffe-Burton and Wakenshaw, 2011). Social identity, group norm, and social influence also positively moderate the links between flow experience and online shopping intention (Hyun et al., 2022). These suggest an open question if the findings observed in our study could be extended to other groups, and further studies are needed to explore it.

Another possibility could be that SES, indeed, modulates shopping behaviors and experience, but its function is subject to the influence of other variables and more complex than the simple linear model. Take purchase intention, for instance, some reported a positive relationship between income level and online purchasing intention as higher income-level consumers may believe that shopping online would save them time as they may value time for the accrued opportunity cost (Punj, 2012). However, a reverse scenario is also reported that online consumers in Beijing who have high-income levels do not tend to shop online because they prefer buying branded products at retail stores in order to have a nice user experience, support, and service (Clemes et al., 2014).

Consistent with the latter speculation, though we did not find a significant main effect of SES on flow experience, we observed an interaction between SES and level of telepresence on flow experience during online shopping of female college students: telepresence demonstrated a smaller impact on flow experience in respondents with lower subjective rating of SES, but larger impact in those with higher feeling of SES. In other words, those in higher SES have less flow experience than the low-SES individuals when the level of telepresence is low, but experience higher experience of flow than the low-SES individuals when telepresence is at a high level. Meanwhile, our study showed that socioeconomic status moderated the mediation of flow experience, while website telepresence influenced purchase intention through the mediation of flow experience, and the mediating effect was smaller in women with lower socioeconomic status than those with higher socioeconomic status. These results deliver two pieces of information: first, socioeconomic status, indeed, contributes to a buyer’s shopping experience, and second, it suggests that the effect of socioeconomic status or demographic variables in general on consumer behavior should be scrutinized with more care.

The ubiquity of internet access has leveled the retail playing field, making it easy for individuals and businesses to sell products without geographic limitations. This study shows that website telepresence, characterized mainly by its vividness and interactivity, is beneficial for flow experience and enhances purchase intention, especially for Chinese female college students with high socioeconomic status. The results are implicative for e-commerce businesses at least in two aspects, such as how the websites should be designed, and how the product should be promoted. First, website telepresence improvement is important, for e-vendors targeting whatever stratum of customers. Our study observed a positive association between telepresence and purchase intention in both low and high SES consumers. This suggests the commonness: internet has become a marketplace for all walks of consumers and there are some principles that apply to all of them during online shopping. The enhancement could involve improvement in the running speed of servers, the availability and usability of navigation system information, and so on. E-merchants should not only improve the presentation of product information but also pay attention to enhancing the interaction between consumers and shopping websites. Second, telepresence improvement is particularly important for those e-industries targeting the high SES consumers. The moderated mediation involving socioeconomic status identified in our study means that other than “turn attention away from socioeconomic variables” (Hernández et al., 2011), it is necessary for the e-merchants to formulate sale strategies and target consumers based on socioeconomic characteristics such as income and education levels. For e-venders targeting high SES customers, they should also not allow low levels of telepresence on their websites in order to avoid a more intensively negative shopping experience. Our results can also be informative for the vendors to formulate product promotion. Admittedly, it is important to ensure all customers have equal legal rights to get access to product information, but when promoting the products, the e-merchants or sale agents can adjust their emphasis on product features, hedonic aspects, or utilitarian aspects, to meet the needs emphasized differently by customers from a variety of backgrounds.

## Limitations and suggestions for future study

Readers are suggested to be aware of several limitations in the current study. First, as our participants were limited to female college students, it is unknown if our results could generalize to other female groups, for instance, those who have graduated and are now working or married. To improve the external validity of the study, other female groups should be included in future studies. Second, this study only considered socioeconomic status as the consumer characteristics of interest. Future research can continue to explore the moderation of other individual factors. In addition, the present paper only focuses on purchase intention but there are many forms of online shopping, so further studies are merited to explore the relationship between the flow experience and other variables of online purchasing such as impulse purchases.

## Conclusion

Our study, based on a group of female Chinese college students, observes the positive effect of website telepresence on purchase intention, and the mediation of flow experience behind this relationship. The mediation of flow experience is further moderated by socioeconomic status as higher socioeconomic status is linked to stronger the mediation effect of flow experience.

## Data availability statement

The raw data supporting the conclusions of this article will be made available by the authors, without undue reservation.

## Ethics statement

The studies involving human participants were reviewed and approved by the Ethics Committee of Chongqing Normal University. The patients/participants provided their written informed consent to participate in this study.

## Author contributions

GZ: conceptualization, funding acquisition, investigation, original draft, reviewing and editing, and significant revision. SJ: conceptualization, data analysis, significant revision, and reviewing and editing. KL: investigation, data analysis, and original draft. All authors contributed to the article and approved the submitted version.

## Footnotes

1
https://www.cnki.net/
