# Supplementary material for: A moderated mediation mechanism underlying the impact of website telepresence on purchase intention — Evidence from Chinese female college student customers
Source: Front Psychol. 2022 Sep 2;13:902414. doi: 10.3389/fpsyg.2022.902414 (PMC9478893; doi:10.3389/fpsyg.2022.902414)
Supplement: Supplementary file 1 [file Presentation_1.pdf]

Supplementary Materials

# A moderated mediation mechanism underlying the impact of website telepresence on purchase intention — evidence from Chinese female college student customers

Guiqin Zhu, Shuaihe Jiang* and Kai Li

Frontiers in Psychology

10.3389/fpsyg.2022.902414

## Appendix 1: Questionnaires

We are a researcher at ** University, which is a survey on online shopping for female college students. Please read the following questions carefully and answer according to the facts about you. The data of this survey is only for scientific research, we will keep your personal information and data strictly confidential. please feel free to answer. We are deeply grateful for your participation!

1. How thoroughly and carefully did you browse the web?
☐Very unserious ☐not serious ☐less serious ☐more serious ☐more serious ☐more serious
2. The brand you just viewed is: ______
3. Your major: ☐ Liberal Arts ☐ Sciences ☐Engineering ☐Sports ☐ Arts
4. Your grade: ☐Freshman ☐Sophomore ☐Year 3 ☐ Year 4 ☐ Master Candidates
5. Your age: ______
6. Your monthly living expenses are: ________RMB
7. Years of experience in online shopping: ________ Years

Thank you for completing the experimental task as required. Please answer the following questions according to the most direct experience and feeling when you browsed the website just now, and mark "√" on the most suitable option. 1-7 represents the following in order: totally disagree, disagree, somewhat disagree, not sure, somewhat agree, agree and totally agree.

**Website telepresence scale** (Fiore et al., 2005)

| Items | Totally disagree | disagree | somewhat disagree | not sure | somewhat agree | agree | totally agree |
|---|---|---|---|---|---|---|---|
| 1. The website makes it easy for me to understand the real style of the product. | 1 | 2 | 3 | 4 | 5 | 6 | 7 |
| 2. The website provides me with information (size, color, details, etc.) that I can get in physical stores. | 1 | 2 | 3 | 4 | 5 | 6 | 7 |
| 3. The website provides me with a shopping experience similar to that of a physical store. | 1 | 2 | 3 | 4 | 5 | 6 | 7 |
| 4. The website allows me to interact (e.g. click and read buyer reviews). | 1 | 2 | 3 | 4 | 5 | 6 | 7 |
| 5. The website provides me with accurate product information. | 1 | 2 | 3 | 4 | 5 | 6 | 7 |

**Online Purchase Intention Scale** (C. Wang et al., 2017)

| **Item** | Totally disagree | disagree | somewhat disagree | not sure | somewhat agree | agree | totally agree |
|---|---|---|---|---|---|---|---|
| 1. If I need to buy clothing online, I will consider buying it on this website. | 1 | 2 | 3 | 4 | 5 | 6 | 7 |
| 2. If I need to buy clothing online, I am willing to use this website to purchase. | 1 | 2 | 3 | 4 | 5 | 6 | 7 |
| 3. I would like to recommend this website to my friends. | 1 | 2 | 3 | 4 | 5 | 6 | 7 |

**Flow experience scale** (Webster et al., 1993)

| Item | totally disagree | disagree | somewhat disagree | not sure | somewhat agree | agree | totally agree |
|---|---|---|---|---|---|---|---|
| 1. The interaction with the website made me curious. | 1 | 2 | 3 | 4 | 5 | 6 | 7 |
| 2. I feel like I'm in control when I'm browsing the website. | 1 | 2 | 3 | 4 | 5 | 6 | 7 |
| 3. There is an inherent pleasure in browsing the website. | 1 | 2 | 3 | 4 | 5 | 6 | 7 |
| 4. The site allows me to control the interaction with the computer. | 1 | 2 | 3 | 4 | 5 | 6 | 7 |
| 5. Browsing the website sparked my curiosity. | 1 | 2 | 3 | 4 | 5 | 6 | 7 |
| 6. Browsing the website bored me. | 1 | 2 | 3 | 4 | 5 | 6 | 7 |
| 7. I am engrossed in navigating the website. | 1 | 2 | 3 | 4 | 5 | 6 | 7 |
| 8. When browsing the website, I realize that I am distracted. | 1 | 2 | 3 | 4 | 5 | 6 | 7 |
| 9. I was thinking of something else while browsing the website. | 1 | 2 | 3 | 4 | 5 | 6 | 7 |
| 10. This website is interesting to me. | 1 | 2 | 3 | 4 | 5 | 6 | 7 |
| 11. Browsing the website evoked my imagination. | 1 | 2 | 3 | 4 | 5 | 6 | 7 |
| 12. I feel like I can't control my interaction with the website. | 1 | 2 | 3 | 4 | 5 | 6 | 7 |

**Socioeconomic Status Rating (Adler et al., 2000)**

*Please see the following drawing of a ladder with 10 rungs and read the following instruction:*

"Imagine that this ladder pictures how Chinese society is set up. At the top of the ladder are the people who are the best off — they have the most money, the highest amount of schooling, and the jobs that bring the most respect. At the bottom are people who are the worst off — they have the least money, little or no education, no job, or jobs that no one wants or respects. Now think about your family. Please tell us where you think your family would be on this ladder. Mark the rung that best represents where your family would be on this ladder."

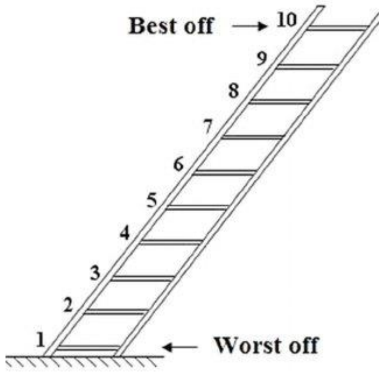

# Appendix 2

**Websites with high telepresence**

The websites with high telepresence in our study have higher interactivity, higher vividness. For instance, they ensure the customers that the products could be returned back and refund if the customers are not satisfied. They also allow the customers to give comments, and get access to comments and feedbacks from previous buyers.

**Websites with low telepresence**

The websites wih low telepresence in our study, in contrast, present only information such as the images, prices, the available sizes. The apparels are presented from fewer perspectives than in the high-telepresence websites. They do not ensure the return if unsatisfied, it do not allow the customers to give their comments, or get access to comments and feedbacks from previous buyers either.

# Appendix 3 Codes and Results of Structural Equation Modelling in Mplus

```
title: mod med;
data: file is datafrontiers2.dat;
vari: NAMES ARE g1 g2 g3 g4 m1 m2 zx zy zm zw zc1 zc2 zc3 zc4;
usev= g1 g2 g3 g4 m1 m2 zx zy zm zw zc1 zc2 zc3 zc4 int;

DEFINE:  int = zx * zw;
standardize int;

analysis: boot = 5000;

model:
zm on zx(beta1)
zw(beta2)
int(beta3)
zc1
zc2
zc3
zc4
g1
g2
g3
g4
m1
m2;

zy on zx(beta4)
zm(beta5)
zw
int
zc1
zc2
zc3
zc4
g1
g2
g3
g4
m1
m2;

model constraint:
new(med slp_hi slp_lo med_hi med_lo);
med = beta1 * beta5;
slp_hi = beta1 + beta3;
slp_lo = beta1 - beta3;
med_hi = slp_hi * beta5;
med_lo = slp_lo * beta5;

output: stand cint(bcboot);
```

MODEL RESULTS

```
                                                    Two-Tailed
                    Estimate       S.E.  Est./S.E.    P-Value

 ZM       ON
    ZX                 0.556      0.072      7.735      0.000
    ZW                 0.012      0.063      0.193      0.847
    INT                0.189      0.061      3.077      0.002
    ZC1                0.118      0.076      1.553      0.120
    ZC2                0.316      0.072      4.362      0.000
    ZC3               -0.010      0.110     -0.089      0.929
    ZC4               -0.086      0.062     -1.382      0.167
    G1                -0.028      0.273     -0.102      0.918
    G2                -0.328      0.265     -1.237      0.216
    G3                 0.070      0.318      0.221      0.825
    G4                -0.080      0.323     -0.249      0.803
    M1                 0.191      0.492      0.388      0.698
    M2                 0.213      0.472      0.452      0.651

 ZY       ON
    ZX                 0.452      0.093      4.860      0.000
    ZM                 0.376      0.097      3.872      0.000
    ZW                 0.028      0.067      0.417      0.677
    INT                0.088      0.063      1.409      0.159
    ZC1                0.039      0.072      0.550      0.583
    ZC2               -0.042      0.078     -0.536      0.592
    ZC3                0.043      0.118      0.361      0.718
    ZC4               -0.019      0.075     -0.248      0.804
    G1                 0.349      0.246      1.417      0.157
    G2                 0.158      0.262      0.606      0.545
    G3                 0.148      0.295      0.503      0.615
    G4                 0.164      0.321      0.513      0.608
    M1                 0.846      0.269      3.142      0.002
    M2                 0.866      0.255      3.400      0.001

 Intercepts
    ZY                -0.970      0.326     -2.970      0.003
    ZM                -0.126      0.525     -0.240      0.810

 Residual Variances
    ZY                 0.374      0.043      8.679      0.000
    ZM                 0.402      0.045      8.906      0.000

 New/Additional Parameters
    MED                0.209      0.066      3.168      0.002
    SLP_HI             0.745      0.091      8.166      0.000
    SLP_LO             0.367      0.098      3.756      0.000
    MED_HI             0.280      0.087      3.209      0.001
    MED_LO             0.138      0.055      2.506      0.012
```
